# The Worksite Health Promotion Capacity Instrument (WHPCI): development, validation and approaches for determining companies' levels of health promotion capacity

**DOI:** 10.1186/1471-2458-10-550

**Published:** 2010-09-13

**Authors:** Julia Jung, Anika Nitzsche, Melanie Neumann, Markus Wirtz, Christoph Kowalski, Jürgen Wasem, Brigitte Stieler-Lorenz, Holger Pfaff

**Affiliations:** 1 Institute for Medical Sociology, Health Services Research and Rehabilitation Science (IMVR), Faculty of Human Sciences and Medical Faculty, University of Cologne & Centre for Health Services Research Cologne (ZVFK), Cologne, Germany; 2 Medical Department of the University of Witten/Herdecke, Integrated Curriculum for Anthroposophic Medicine (ICURAM), Herdecke, Germany; 3 Institute for Psychology, University of Education Freiburg, Freiburg, Germany; 4 Chair of Medical Management; University of Duisburg-Essen, Essen, Germany; 5 Core Business Development GmbH, Berlin, Germany

## Abstract

**Background:**

The Worksite Health Promotion Capacity Instrument (WHPCI) was developed to assess two key factors for effective worksite health promotion: collective willingness and the systematic implementation of health promotion activities in companies. This study evaluates the diagnostic qualities of the WHPCI based on its subscales Health Promotion Willingness and Health Promotion Management, which can be used to place companies into four different categories based on their level of health promotion capacity.

**Methods:**

Psychometric evaluation was conducted using exploratory factor and reliability analyses with data taken from a random sample of managers from n = 522 German information and communication technology (ICT) companies. Receiver operating characteristic (ROC) analyses were conducted to determine further diagnostic qualities of the instrument and to establish the cut-off scores used to determine each company's level of health promotion capacity.

**Results:**

The instrument's subscales, Health Promotion Willingness and Health Promotion Management, are based on one-dimensional constructs, each with very good reliability (Cronbach's alpha = 0.83/0.91). ROC analyses demonstrated satisfactory diagnostic accuracy with an area under the curve (AUC) of 0.76 (SE = 0.021; 95% CI 0.72-0.80) for the Health Promotion Willingness scale and 0.81 (SE = 0.021; 95% CI 0.77-0.86) for the Health Promotion Management scale. A cut-off score with good sensitivity (71%/76%) and specificity (69%/75%) was determined for each scale. Both scales were found to have good predictive power and exhibited good efficiency.

**Conclusions:**

Our findings indicate preliminary evidence for the validity and reliability of both subscales of the WHPCI. The goodness of each cut-off score suggests that the scales are appropriate for determining companies' levels of health promotion capacity. Support in implementing (systematic) worksite health promotion can then be tailored to each company's needs based on their current capacity level.

## Background

Worksites are viewed as an effective setting for health promotion [1-3]. Not only are worksite conditions associated with employee health and well-being, it is the worksite where most people spend the majority of their time and can be reached by health promotion activities. However, not all companies implement comprehensive worksite health promotion (WHP), and even if they do, it is not always supported by a management process [4-10]. The result is a great need to establish WHP in more companies and to ensure that it is being implemented systematically. When seeking to assist those companies that have not yet established (systematic) WHP, the problem quickly encountered is that not all companies possess the same capacity for engaging in WHP. Each requires support tailored to their specific level of capacity.

The present study introduces the new Worksite Health Promotion Capacity (WHPC) model, which is based on several other approaches to measuring and defining health promotion [e.g. [11-13]]. Similar to authors of studies on the capacity for heart health promotion, which look at factors such as will and an extensive infrastructure as being key in health promotion efforts [11,14,15], the authors of this study developed a WHPC model that takes into account the willingness of a company to implement WHP and the existence of a management process for ensuring its successful implementation. By measuring these two dimensions, it is then possible to place companies into four different categories based on their level of health promotion capacity. The model builds on the results of a qualitative study on the use of and conditions for WHP. In the study, an organization's "health promotion willingness" is defined as the willingness of a company to implement WHP on a permanent basis. The second dimension of the model, "health promotion management," involves the extent to which WHP is being put into practice systematically in the form of a management process (problem-solving cycle) or a health promotion program [16]. Only once these conditions have been met, does a company possess the highest level of capacity and the fundamental requirements for effective and sustainable worksite health promotion [17].

**Figure 1:**
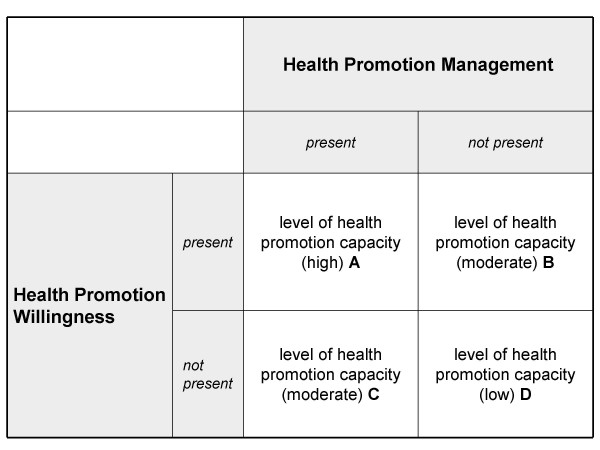
**Categorization of companies according to level of health promotion capacity**.

These two dimensions are measured and used to determine a company's level of health promotion capacity as described below (see also Figure 1):

• Level A companies are characterized by the willingness, particularly at the highest executive management level, to reduce work-related stress and strengthen health resources (i.e., to promote the health of employees) and by the existence of a system for managing health promotion activities. These companies are considered to have a high level of capacity for engaging in health promotion.

• Level B companies have a limited health promotion capacity. These companies possess the willingness to implement worksite health promotion but (still) do not have the appropriate management system for implementing it. The health promotion capacity of these companies is moderate.

• Level C companies also have a limited capacity for engaging in worksite health promotion. These companies have the proper management system in place but have lost some of the willingness to promote employee health, perhaps as a result of a change in management or the occurrence of certain obstacles. Like Level B companies, Level C companies also have a moderate level of health promotion capacity.

• Level D companies have neither the willingness nor an appropriate management system in place for engaging in worksite health promotion. The health promotion capacity of these companies is low.

As previously mentioned, it is important to determine each company's particular situation in terms of their capacity to engage in WHP. By categorizing companies according to level of capacity, we establish a framework for providing each company with support targeted at the particular phase they are in the establishment of systematic WHP rather than simply providing them with "one-size-fits-all" solutions.

The objective of this exploratory study is to introduce an economical and time-saving instrument for determining the health promotion capacity of companies. The study also makes first attempts to test the psychometric quality of the Worksite Health Promotion Capacity Instrument (WHPCI) and to establish cut-off scores for each of the instruments' subscales, Health Promotion Willingness and Health Promotion Management. The cut-off scores are then used to determine companies' levels of health promotion capacity.

## Methods

### Study sample

Data for evaluating the WHPCI were taken from a cross-sectional study conducted within the Prevention Competence Network (PraeKoNet) Project. The study, which was approved by the Ethics Committee of the University Hospital of Cologne, aimed to examine, inter alia, the state of WHP within the German information and communication technology (ICT) industry in 2008. The target population was all companies in the ICT industry with ten or more employees. The address database of Schober Group International http://www.schober.com, which had been identified as being the largest and most extensive database for companies in Germany, was used to obtain a representative sample of the target population. A sample of companies was randomly selected from each of three size categories. This categorization is based on the recommendation of the European commission to classify large companies as having 250 or more employees, medium-sized companies as having between 50 and 249 employees and small companies as having between 49 and 10 employees [18]. Each sample company was then randomly assigned to an interview list so that each list would constitute a valid probability sample. The sample in this study was drawn disproportionately with respect to company size.

**Figure 2:**
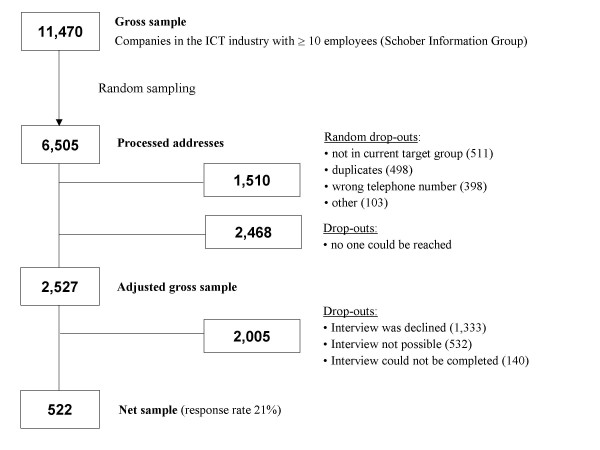
**Flow chart illustrating the sampling procedure**.

Telephone interviews were conducted until a total number of at least 500 interviews had been completed. To ensure the highest response rate, a letter of introduction was sent [19] and up to 15 contact attempts were made. Interviews were conducted with one managing director (or a representative appointed by him/her) (see Table 1) from each company. The decision to interview managing directors as key organizational informants was made on the assumption that they are most familiar with the structures and processes of the company. This strategy of interviewing one key person from each organization is also an accepted method of organizational research [20]. An additional reason for speaking with managing directors is that they are the main drivers behind WHP [11,15,21-25]. In the end, it was possible to conduct interviews with key informants from 522 companies, giving a response rate of 21% (see Figure 2).

**Table 1:** Positions of the company representatives interviewed (n = 522)

Job title	n	%
owner, proprietor	37	7.1
managing director, member of the board of directors	168	32.2
head of division, senior department head	117	22.4
department head	85	16.3
assistant to executive management	37	7.1
human resources manager/director	51	9.8
Other	27	5.1

The resulting disproportionate sample was composed of 223 small (42.7%), 171 medium-sized (32.8%) and 128 large companies (24.5%) in the German ICT industry as opposed to the sample population in which 81% of companies were small, 14% were medium-sized and approximately 5% were large.

### Measures

To guarantee the content validity of the instrument and to ensure that the instrument's dimensions are relevant to current practice, two steps were taken. First the instrument's two dimensions, health promotion willingness and health promotion management, were operationalized based on international guidelines and previous research. In the next step, the study's findings were discussed in a focus group of organizational experts (n = 7) and a consensus emerged on the operationalization of the scales and scale items.

#### The Health Promotion Willingness scale

The health promotion willingness dimension measures the willingness of a company to implement worksite health promotion on a permanent basis. The willingness of a company's main political actors was found to have a key impact on this dimension. At the same time, this willingness must be present at the executive management level [e.g. [11,21,26,27]]. Furthermore, the more influential individuals within the company are not persuaded of the advantages of health promotion and, in fact, work against it (resistance), the more the overall willingness within a company to implement WHP decreases. Moreover, the willingness to establish WHP can only exist if there is an initial positive collective expectation (outcome expectation) regarding WHP and if there is a common conviction that WHP will be beneficial for the company's employees and/or the company as a whole. This aspect of health promotion willingness is in line with the findings of other studies, which suggest that the willingness to adopt an innovation is determined by beliefs and attitudes towards the innovation [12,28]. In addition, an assumption of the qualitative results is that within a company there must prevail a collective opinion that the company has the capacity to successfully initiate and implement WHP (collective efficacy). Both components - outcome expectation and efficacy - show similarities to Bandura's social learning theory, whereby an intent to change individual behavior is then probable when the person has a high level of self-efficacy and a positive outcome expectation regarding the change in behavior [29]. Although this theory pertains to the individual level, the experts participating in the focus group found aspects of the theory relevant to willingness at the organizational level as well, as was found by another study [14]. Another element of the collective willingness to implement WHP was seen as the extent to which the company views the health of its employees as its responsibility. A further indication of the existence of willingness is the degree to which the issue of health promotion is discussed within the company. We consider this an outward expression of the collective willingness to engage in WHP. By making health promotion a topic of discussion, the acceptance of WHP also becomes a norm within the company.

Based on these considerations, six items were originally developed for the Health Promotion Willingness scale [Additional file 1].

#### The Health Promotion Management scale

The second dimension of the model, health promotion management, encompasses the extent to which WHP is systematically conceived, controlled and organized in the form of a management process (problem-solving cycle) or a health promotion program [11,30-32]. Similar approaches to measuring health promotion capacity based on whether an organization implements health promotion and prevention activities systematically were used in the studies of Riley et al. [12] and Schwartz et al. [13] and are also found in European WHP guidelines and quality criteria [27]. Implementing health promotion activities and measures systematically ensures effective and sustainable WHP [33,34].

The Health Promotion Management subscale originally consisted of five items [Additional file 1].

The items of both subscales were each measured on an eleven-point scale ranging from "do not agree at all" to "agree completely" and are coded in such a way that a higher value indicates a positive assessment by the respondent (10) and a lower value indicates a negative assessment (0).

Further development of the instruments involved conducting qualitative pre-tests with a sample of n = 10 managing directors or decision-makers using cognitive approaches, such as the think-aloud method and the probing technique [35].

Apart from using the two WHPCI scales, the survey of companies also collected data on other relevant WHP quality criteria and key corporate indicators in order to obtain a more detailed picture of the current state of WHP in ICT companies. First, the extent to which WHP measures were being implemented in the companies was measured using five items (e.g., *"Our company offers a variety of measures for changing employee health behavior."*). Once again, each item was measured on an eleven-point rating scale. If a sum score ("activ_sum") of 0 was recorded -- i.e., no interventions of any kind were being implemented -- the values of the Health Promotion Management scale items were automatically coded as 0 because it could be assumed that a WHP management process did not exist.

The external criteria used to identify the subscales' cut-off scores, which are then used to determine companies' levels of health promotion capacity, were derived from an instrument that started out with a statement inquiring about the existence of worksite health management within the company ("*Our company has a worksite health management (WHM) program.") *(This statement was preceded by a comprehensive definition of WHM, in which it was explicitly stated that a WHM program must be based on a systematic management process [36-38]). The WHM variable was measured dichotomously (yes = 1; no = 2) and was used to identify the cut-off scores for differentiating between companies in which Health Promotion Management was and was not present (see Figure 1).

This was followed by statements aimed at measuring the companies' willingness to implement WHM ("*Our company is/is not planning to implement WHM in the next three years*.") or to maintain a current WHM program (*"The willingness to continue with WHM in future is/is not (as) present as before*."). These categories were used to construct the dichotomous variable "will_Worksite Health Management," with 0 indicating that willingness was present and 1 indicating that willingness was not present.. The dichotomization made it possible to use an ROC analysis to empirically establish the cut-off scores for differentiating between the two groups "Health Promotion Willingness present" and "Health Promotion Willingness not present" (see Figure 1).

### Data analysis

First, a missing data analysis was performed on the WHPCI items, after which five cases had to be excluded from further analysis because values were missing for ≥ 30% of the items. In the next step, all other missing item values were imputed to prevent biases caused by non-random missing values. Imputation was performed in the NORM software program using a widely accepted procedure based on the Expectation Maximization (EM) algorithm [39]. Through an iterative process involving the maximum likelihood method, missing item values were estimated based on the covariance matrix of the observed parameters. Maximum plausible values were then imputed, while taking random variability into account [39].

In the case of n = 12 companies, all item values of the Health Promotion Management scale were automatically coded as 0 because the companies had a sum score of 0 for the WHP measures scale.

#### Exploratory factor and reliability analyses

In keeping with traditional test theory, an exploratory factor analysis (EFA) using varimax rotation was performed on the items of both subscales to identify their underlying latent factors. Factor loadings above 0.50 were considered salient [40]. Based on the results of the EFA, a reliability analysis (RA) was conducted to determine the internal consistency of the scales. The Cronbach's alpha (threshold ≥0.70) was then examined [40].

#### Receiver operating characteristic analyses

Based on the results of the EFA and RA, cut-off scores were identified for both scales. These cut-off scores represent a specific value of the total sum score of each scale and are used to distinguish between companies with or without health promotion willingness and health promotion management. Ultimately, the scores help to place companies into one of the four categories of health promotion capacity.

The receiver operating characteristic (ROC) analysis is an accepted procedure used to identify a cut-off for differentiating between two groups with the help of an external criterion [41,42]. The sensitivity (true positive rate) and specificity (true negative rate) of each potential cut-off are then calculated [43,44]. We chose cut-offs that demonstrate a maximum Youden Index Y (sensitivity + specificity - 1) in order to obtain a good trade-off between false-positive and false-negative decisions in one step [42,44].

Furthermore, in an ROC analysis, the area under the curve (AUC) is calculated to determine the diagnostic value of a test [41]. A test with an AUC of 0.50 is seen as having poor diagnostic accuracy, whereas a test with an AUC of 1.0 is considered to have perfect diagnostic accuracy [41,44]. Non-medical procedures with values of 0.65-0.70 are considered good [41,45]. Further assessments of the criterion validity of the test, in particular its predictive validity, are based on the sensitivity and specificity of the identified cut-offs [45,46].

#### Logistic regression analyses

Logistic regression analyses (LRAs) were used to assess the predictive power of the cut-off scores of the Health Promotion Willingness and Health Promotion Management scales. ROC analyses and LRAs can be regarded as complementary techniques. Using LRAs, we examined whether each scale's cut-off score adequately predicts the incidence of the respective external criterion. We also calculated the efficiency of each scale, i.e. the percentage of all cases and non-cases being correctly classified by each scale.

All calculations were performed using the Software Statistical Package for Social Science (SPSS) Version 16. P-values < 0.05 were considered significant.

**Ethical approval**: Yes

## Results

### Descriptive findings

To supplement data from the psychometric evaluation, the descriptive statistics of both subscales of the WHPCI and their items are provided in [Additional file 1]. The number of missing values for all WHPCI items did not exceed the threshold of 5% warranting possible elimination [47].

Willingness to actively engage in WHP is only present to a rather moderate extent at the management level. However, only few were found to be working against it. Employee health is not only considered to be the responsibility of employees themselves; the company too is seen as sharing in the responsibility to a somewhat equal degree. The topic of health promotion is, however, relatively rarely discussed and companies possess rather limited efficacy expectation (Item 5). Nevertheless, companies seem to have a relatively high outcome expectation.

There is still a considerable need for companies to implement WHP systematically. So far, very few companies have done so, either in the form of a management process or bundled in a company health promotion program (Items 7-11).

The descriptive results of the external criteria for the ROC analyses are presented in Table 2. Almost two-thirds of companies stated that they do not implement WHM. Nearly one-half of the companies surveyed indicated that either they were not willing to implement it within the next three years or they were no longer (very) willing to maintain an existing WHM program.

**Table 2:** Descriptive statistics of the external criteria for the receiver operating characteristic analysis

Instrument	n	**present **(1)		**not present **(0)	
		**n**	**%**	**n**	**%**
will_Worksite Health Management	508	224	44.1	284	55.9
Worksite Health Management	519	135	26.0	384	73.0

#### Exploratory factor and reliability analyses

The initial EFA, which was performed with all items, extracted two factors. Only Item 2 did not achieve the necessary factor loading [Additional file 2] (Model 1) and was consequently eliminated. Consistent with our theoretical assumptions, all other items loaded on one of the two factors that had been extracted.

A joint EFA was again performed [Additional file 2] (Model 2) without Item 2, and separate EFAs were conducted on each subscale [Additional file 2] (Model 3). The EFA with only the items of the Health Promotion Willingness scale extracted a factor with an eigenvalue of 2.95 (Kaiser's criterion), which accounted for 59.1% of the total variance. The EFA of the Health Promotion Management scale items revealed a single-factor solution with an eigenvalue of 3.76 and a total explained variance of 75.1%.

As a result of the RA of the Health Promotion Willingness scale, Item 3 had to be eliminated because its item total correlation did not achieve the threshold value of 0.50 [Additional file 2]. Removing Item 3 improved Cronbach's alpha from 0.82 to 0.83. With regard to the internal consistency of the Health Promotion Management scale, a Cronbach's alpha of 0.91 was achieved without having to eliminate any items.

A bivariate correlation analysis of both scales produced a Pearson's coefficient of r = 0.56 (p < .001).

#### Determination of sensitive and specific cut-off scores

**Figure 3:**
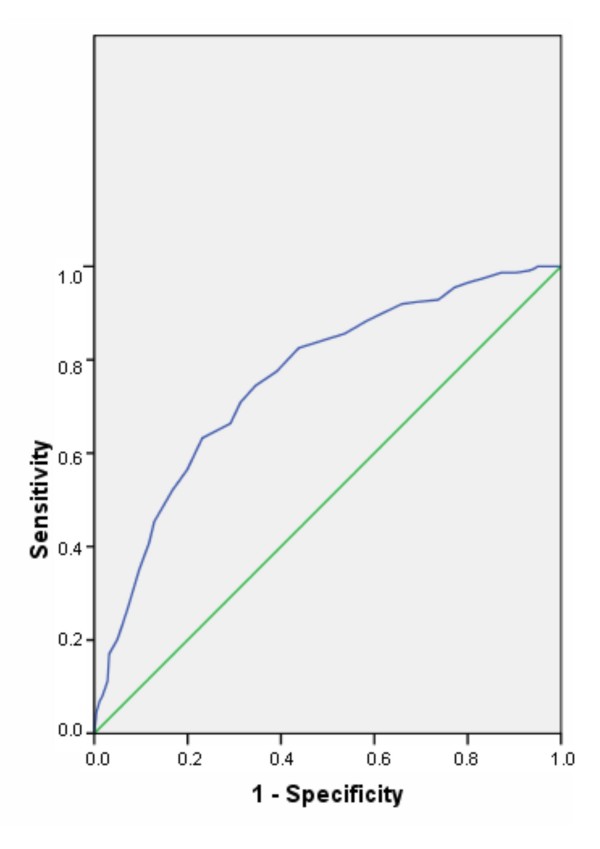
**Receiver operating characteristic (ROC) curves of the Health Promotion Willingness scale**.

The ROC analysis of the Health Promotion Willingness scale resulted in an area under the curve (AUC) of 0.76 (SE = 0.021; p < 0.001; 95% CI 0.72-0.80). A Youden Index Y = 1 was calculated for a cut-off of 6. The sensitivity of this cut-off is 71% and the specificity is 69% (see also Table 3 and Figure 3). All cases with a mean scale score of < 6 were classified as "Health Promotion Willingness not present" (0); all others were classified as "Health Promotion Willingness present" (1). With a total of 70% correct classifications (i.e., true positives + true negatives), the scale exhibited good efficacy and proved to have good predictive power.

**Table 3:** Classification of companies with and without health promotion willingness and the external criterion will_Worksite Health Management

		Health Promotion Willingness		percentage of correct classifications
		**not present (n)**	**present (n)**	**%**

**will_Worksite Health Management**	**not present**	193	88	69.0
	**present**	65	158	71.0
**total percentage**				70.0

**Figure 4:**
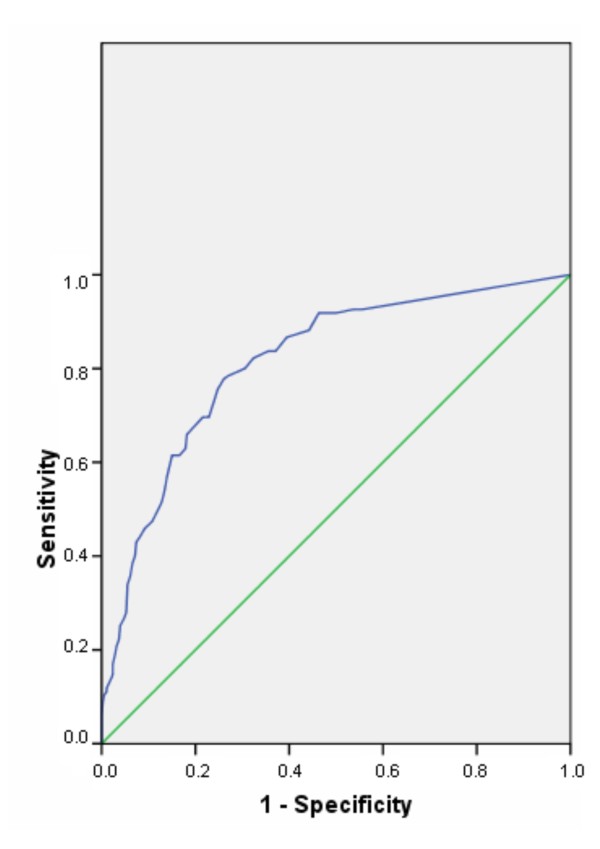
**Receiver operating characteristic (ROC) curves of the Health Promotion Management scale**.

The Health Promotion Management scale was found to have an AUC of 0.81 (SE = 0.021; p < 0.001; 95% CI 0.77-0.86). A Youden Index Y = 1 was achieved with a cut-off score of 2.55, giving a sensitivity of 76% and a specificity of 75% (see also Table 4 and Figure 4). This means that all cases with a mean scale score of ≤ 2.55 are classified as "Health Promotion Management not present" (0); all others are classified as "Health Promotion Management present" (1). The scale exhibited good efficacy with a total of 75% correct classifications and proved to have good predictive power.

**Table 4:** Classification of companies with and without health promotion management and the external criterion Worksite Health Management

		Health Promotion Management		percentage of correct classifications
		**not present (n)**	**present (n)**	**%**

**Worksite Health Management**	**not present**	286	94	75.0
	**present**	33	102	76.0
**total percentage**				75.0

The cut-off scores of the Health Promotion Willingness scale and the Health Promotion Management scale were entered as predictors into two separate LRAs with the respective external criteria to further examine each scale's predictive power. The odds ratio (OR) for the Health Promotion Willingness scale was 5.33 (SE = 0.19; p ≤ 0.001; 95% CI 3.63-7.82) and 9.4 (SE = 0.23; p ≤ 0.001; 95% CI 5.96-14.85) for the Health Promotion Management scale.

517 companies could be placed into one of the categories of health promotion capacity. Of these, 154 (29.8%) were categorized as Level A companies and 99 (19.1%) were categorized as Level B companies. By contrast, very few companies (8.3%; n = 43) were categorized into Level C. The majority of companies (42.7%; n = 221) fell into the Level D category of health promotion capacity.

## Discussion

The objective of this study was to psychometrically evaluate the newly developed Worksite Health Promotion Capacity Instrument (WHPCI) along with its two subscales, Health Promotion Willingness and Health Promotion Management, and to consequently produce a valid and diagnostically accurate instrument for categorizing companies into four levels of health promotion capacity using statistical methods.

The main finding of this study was that the EFAs produced one-dimensional solutions for both the Health Promotion Willingness and Health Promotion Management scales. Although during the qualitative development phase of the instrument items 2 and 3 were considered important for a company's willingness to implement WHP, both items had to be removed from the Health Promotion Willingness scale because they did not reach the factor loading or item total correlation cut-offs [Additional file 2] (Models 1 and 2). It may be that the aspect of resistance to health promotion efforts is less relevant than the other components of health promotion willingness. By changing the wording of item 3, which examined the company's share of responsibility in employee health, it may be possible to achieve improved results.

The results of the RAs show that both scales of the WHPCI can be considered as having high internal consistency [40]. As originally theorized, the bivariate analysis found a significant correlation between the scales and their respective constructs. However, this does not necessarily mean that the strength of this correlation should be attributed to congruence.

ROC analyses were conducted to determine the accuracy of the WHPCI scales when measuring health promotion willingness and health promotion management in companies and to identify cut-off scores for placing companies into one of four categories of health promotion capacity: A (high capacity), B and C (moderate capacity) or D (low capacity). The results of the ROC analyses showed that the accuracy (AUCs) of both scales was significantly higher than that of random classification and that the calculated AUCs of both scales were good [41,45]. This implies that the scales could discriminate well between organizations with and without health promotion willingness and health promotion management.

Cut-off scores for each scale were then each defined as the value of the total sum score that produced an optimal balance between sensitivity and specificity (Youden Index Y). The cut-off scores identified for both scales were found to have good sensitivity and specificity [41,45]. Both scales provided good predictive power and exhibited good efficiency.

### Strengths and limitations

Although there were several limitations to this study, the study also has several noteworthy strengths. Among these are the randomized sampling procedure and the size of the study sample. In terms of response rate, it should be noted that surveys with this type of study design and target group generally result in a high number of drop-outs [19,48]. Therefore, the possibility of non-response bias in our results could not be ruled out. A non-response analysis was conducted to identify any potential differences in the response behavior of the survey participants based on company size, but none were found. Nevertheless, there is still the possibility that selection bias may have resulted in an overestimation of the study's results. Non-respondents may have been less interested in WHP than respondents.

Furthermore, interpretation of the results is limited because our data represent the attitudes and opinions of only one person - a manager or representative - as a key informant from each company. The assessments of other company members may differ from those of the key informants [49].

The cross-sectional design of the present study did not allow us to assess the scales' test-retest reliability, which in turn limits their internal validity. The scales would also benefit from further theoretical development, confirmatory factor analysis and further (divergent and convergent) construct validation, as well as cross-validation in other populations. Due to the elimination of two items from the Health Promotion Willingness scale (see Section 2), further development of the scale would be worthwhile.

Our study assessed the efficacy and outcome expectation of companies with regard to WHP, which could be considered similar to Bandura's social learning theory [50]. However, we were only be able obtain a general measurement of these two aspects. Future studies are warranted to investigate the full complexity of these concepts, their measurement and the applicability of theories of individual behavior change.

As we proceeded with our research, we also noted that the health promotion willingness of companies is a particularly complex and multidimensional concept requiring further study. We do not believe that the components of health promotion capacity that we have identified are exhaustive. Future theoretical research may help to enrich the construct.

## Conclusion

This article provides a very detailed account of the preliminary development and psychometric quality of the WHPCI, which should be expanded upon in future studies. The results documented in this article suggest that the instrument is suitable for use in future studies to diagnose, describe, explain and evaluate the current level of health promotion willingness in companies and the degree to which companies are currently implementing WHP systematically. However, further improvements are needed.

Currently little data is available on worksite health promotion capacity in ICT companies. Use of the WHPCI makes collecting such data possible. The WHPCI also makes it possible to obtain comparable data from other industries (initial studies are already being conducted in cooperation with an employers' liability insurance association), locations or countries. In these studies, it would make sense to take a longitudinal approach and to examine both the sustainability of health promotion measures and their effects on health, social and economic outcomes within the company.

In line with the findings of other studies, the findings of our study provided initial indications of the need for improved health promotion capacity in ICT companies, particularly with respect to the systematic implementation of WHP [4-9]. However, the study was also able to show that these companies have a high outcome expectation with respect to WHP and that it can be utilized as a resource for improving a low level of efficacy expectation or for increasing WHP willingness at the management level. The extremely simple and time-efficient structure of the instrument makes it a practical tool, especially for use in telephone surveys. It can also be used to determine the type of support that should be provided to companies to develop or further develop their systematic implementation of WHP. These approaches are currently being applied in companies as part of the PraeCoNet Project and their feasibility and effectiveness is being tested. Ideally, use of the WHPCI should lead to an improved level of health promotion capacity in B, C and D companies by promoting health promotion willingness and/or a more systematic implementation worksite health promotion. In the case of Level A companies, the instrument should help to optimize the companies' current WHP activities or to stabilize them if a score close to the cut-off score is obtained for the Health Promotion Management scale.

## Competing interests

The authors declare that they have no competing interests.

## Authors' contributions

JJ collected the data, performed the data analysis and wrote the first draft of the manuscript. AN, CK, JW and BSL revised the manuscript for important intellectual content. MN contributed to the editorial review and data analysis. MW contributed to the data analysis and provided technical, statistical and editorial support. HP participated in the design of the study and the editorial review. All authors read and approved the final manuscript.

## Pre-publication history

The pre-publication history for this paper can be accessed here:

http://www.biomedcentral.com/1471-2458/10/550/prepub

## Supplementary Material

Additional file 1 **Descriptive statistics for the two subscales of the Worksite Health Promotion Capacity Instrument and their items**. The file contains a table in which the wording of the instrument's items is presented. Furthermore, the mean and standard deviation of each item and of the scales' sum scores are shown. Click here for file

Additional file 2 **Factor loadings of the items of the Health Promotion Willingness and Health Promotion Management scales**. The file contains a table in which the factor loadings of the items are presented as well as the eigenvalues and the results for the explained variance from three exploratory factor analyses. In addition, the corrected item-total correlation of the reliability analysis is given for each item. Click here for file

## References

[B1] Special Committee on Health PaDM Healthy Workforce/Healthy Economy: The Role of Health Productivity, and Disability Management in Addressing the Nation's health Care Crisis JOEM 2009 51 114 119 19136880 10.1097/JOM.0b013e318195dad2

[B2] European Network for Workplace Health Promotion (ENWHP) Luxembourg Declaration on Workplace Health Promotion in the European Union 2007 http://www.enwhp.org/fileadmin/downloads/press/Luxembourg_Declaration_June2005_final.pdf

[B3] Kuoppala J Lamminpää A Husman P Work Health Promotion, Job Well-Being, and Sickness Absences - A Systematic Review and Meta-Analysis JOEM 2008 50 1216 1227 19001948 10.1097/JOM.0b013e31818dbf92

[B4] Wilson MG DeJoy DM Jorgensen CM Crump CJ Health promotion programs in small worksites: results of a national survey Am J Health Promot 1999 13 358 365 10557508 10.4278/0890-1171-13.6.358

[B5] Linnan L Bowling M Childress J Lindsay G Blakey C Pronk S Results of the 2004 National Worksite Health Promotion Survey Am J Public Health 2008 98 1503 1509 10.2105/AJPH.2006.100313 18048790 PMC2446449

[B6] Plath C Köhler T Krause H Pfaff H Prevention, health promotion and workplace health management in German banks: Results from a nationwide representative survey J Public Health 2008 16 1 9 10.1007/s10389-007-0165-6

[B7] Hollederer A Work-Site Health Promotion in Germany - Results of the IAB-Establishment Panel 2002 and 2004 Gesundheitswesen 2007 69 63 76 10.1055/s-2007-970599 17405078

[B8] Köhler T Janßen C Plath SC Steinhausen S Pfaff H Determinants of Workplace Health Promotion in the Insurance Sector: Results of a Complete Survey of German Insurance Companies in 2006 Gesundheitswesen 2009 71 722 731 10.1055/s-0029-1202784 19431108

[B9] Ulmer J Gröben F Work Place Health Promotion. A longitudinal study in companies placed in Hessen and Thueringen J Public Health 2005 13 144 152 10.1007/s10389-005-0101-6

[B10] Hollander RB Lengermann JJ Corporate characteristics and worksite health promotion programs: Survey findings from Fortune 500 companies Soc Sci Med 1988 26 491 501 10.1016/0277-9536(88)90382-6 3127893

[B11] Hawe P Noort M King L Jordens C Multiplying Health Gains: the critical role of capacity-building within health promotion programs Health Policy 1997 39 29 42 10.1016/S0168-8510(96)00847-0 10164903

[B12] Riley BL Taylor SM Elliott SJ Determinants of implementing heart health promotion activities in Ontario public health units: a social ecological perspective Health Educ Res 2001 16 425 441 10.1093/her/16.4.425 11525390

[B13] Schwartz R Smith C Speers MA Dusenbury LJ Bright F Hedlund S Capacity building and resource needs of state health agencies to implement community-based cardiovascular disease programs J Public Health Pol 1993 14 480 494 10.2307/3342879 8163636

[B14] Anderson D Plotnikoff RC Raine K Cook K Smith C Barrett L Towards the development of scales to measure 'will' to promote heart health within health organizations in Canada Health Promot Int 2004 19 471 481 10.1093/heapro/dah409 15520037

[B15] Dressendorfer RH Raine K Dyck RJ Plotnikoff RC Collins-Nakai RL McLaughlin WK A Conceptual Model of Community Capacity Development for Health Promotion in the Alberta Heart Health Project Health Promot Pract 2005 6 31 36 10.1177/1524839903259302 15574525

[B16] Pfaff H Kuch C Bundesanstalt für Arbeitsschutz und Arbeitsmedizin (BAuA) Präventionsreife, differentielle Prävention und externe Beratung: Kooperationsmodelle für Krankenhäuser Qualität der Arbeit im Gesundheitssektor. Frühjahrstagung der Bundesanstalt für Arbeitsschutz und Arbeitsmedizin. 07. und 08. Juni 2004 2005 Dortmund, Berlin, Dresden: Wirtschaftsverlag NW 119 127

[B17] Pfaff H Präventive Versorgung Präv Gesundheitsf 2006 1 17 23 10.1007/s11553-005-0008-7

[B18] European Commission The new SME definition. User guide and model declaration 2005 http://ec.europa.eu/enterprise/policies/sme/files/sme_definition/sme_user_guide_en.pdf

[B19] Bradshaw L Curran A Eskin F Fishwick D Provision and perception of occupational health in small and medium-sized enterprises in Sheffield, UK Occup Med (Lond) 2001 51 39 44 10.1093/occmed/51.1.39 11235826

[B20] Steckler A Goodman RM Alciati MH Collecting and analyzing organizational level data for health behavior research Health Educ Res 1997 12 i iii 10174214 10.1093/her/12.3.279

[B21] O'Donnell MP Bishop C Kaplan K Benchmarking best practices in workplace health promotion Am J Health Promot 1997 1 1 9

[B22] Sherman B Worksite Health Promotion: A Critical Investment Dis Manag Health Out 2002 10 101 108 10.2165/00115677-200210020-00005

[B23] Della LJ DeJoy DM Goetzel RZ Ozminkowski RJ Wilson MG Assessing management support for worksite health promotion: Psychometric analysis of the leading by example (LBE) instrument Am J Health Promot 2008 22 359 367 10.4278/ajhp.22.5.359 18517097 PMC2743959

[B24] Bradshaw LM Fishwick D Curran AD Eskin F Provision and perception of occupational health in small and medium-sized enterprises in Sheffield, UK Occup Med (Lond) 2001 51 39 44 10.1093/occmed/51.1.39 11235826

[B25] Downey AM Sharp DJ Why do managers allocate resources to workplace health promotion programmes in countries with national health coverage? Health Promot Int 2007 22 102 111 10.1093/heapro/dam002 17339297

[B26] Della LJ DeJoy DM Goetzel RZ Ozminkowski RJ Wilson MG Assessing Managment Support for Worksite Health Promotion: Psychometric Analysis of the Leading by Example (LBE) Instrument Am J Health Promot 2008 22 359 367 10.4278/ajhp.22.5.359 18517097 PMC2743959

[B27] European Network for Workplace Health Promotion (ENWHP) Healthy Employees in Healthy Organisations. Good Practice in Workplace Health Promotion in Europe. Quality Criteria of Workplace Health Promotion. ENWHP 1999 Essen, Federal Association of Company Health Insurance Funds (BKK Bundesverband) Ref Type: Report

[B28] Kim I Kim MI The effects of individual and nursing-unit characteristics on willingness to adopt an innovation. A multilevel analysis Computers in Nursing 1996 14 183 187 8681212

[B29] Bandura A Self-efficacy: Toward a Unifying Theory of Behavioral Change Psychological Review 1977 84 191 215 10.1037/0033-295X.84.2.191 847061

[B30] O'Connor BN The workplace learning cycle. A problem-based curriculum model for the preparation of workplace learning professionals The Journal of Workplace Learning 2004 16 341 349 10.1108/13665620410550312

[B31] World Health Organization (WHO) Regional guidelines for the development of healthy workplaces. Geneva 1999

[B32] Senge PM The fifth discipline. The art and practice of the learning organization 1990 1 New York: Currency Doubleday

[B33] DeJoy DM Wilson MG Organizational Health Promotion: Broadening the Horizon of Workplace Health Promotion Am J Health Promot 2003 17 337 341 12769047 10.4278/0890-1171-17.5.337

[B34] Witte K Managerial Style and Health Promotion Programs Soc Sci Med 1993 36 227 235 10.1016/0277-9536(93)90006-P 8426966

[B35] Prüfer P Rexroth M Verfahren zur Evaluation von Survey-Fragen: Ein Überblick ZUMA-Nachrichten 1996 20 95 116

[B36] Pfaff H Slesina W Effektive betriebliche Gesundheitsförderung. Konzepte und methodische Ansätze zur Evaluation und Qualitätssicherung 2001 Weinheim und München: Juventa

[B37] Chu C Dwyer S Employer role in integrative workplace health management. A new model in progress Dis Manag Health Out 2002 10 175 186 10.2165/00115677-200210030-00005

[B38] Badura B Hehlmann T Betriebliche Gesundheitspolitik. Der Weg zur gesunden Organisation 2003 Berlin, Heidelberg: Springer Verlag

[B39] Schafer JL Graham JW Missing Data: Our View of the State of the Art Psychological Methods 2002 7 147 177 10.1037/1082-989X.7.2.147 12090408

[B40] Hair JF Black WC Babin BJ Anderson RE Multivariate Data Analysis 2009 7 Upper Saddle River: Pearson Education, Inc

[B41] Swets JA Pickett RM Evaluation of Diagnostic Systems. Methods from Detection Theory 1982 Academic Press. Series in Cognition and Perception edn. New York

[B42] Fluss R Faraggi D Reiser B Estimation of the Youden Index and its Associated Cutoff Point Biometrical J 2005 47 458 472 10.1002/bimj.200410135 16161804

[B43] Sackett DL Haynes B Guyatt GH Tugwell P The Interpretation of Diagnostic Data Clinical Epidemiology - A Basic Science for Clinical Medicine 1991 Boston/Toronto/London: Littel, Brown and Company 116 119

[B44] Murphy JM Berwick DM Weinstein MC Borus JF Budman SH Klerman GL Performance of Screening and Diagnostic Tests Arch Gen Psychiat 1987 44 550 555 3579501 10.1001/archpsyc.1987.01800180068011

[B45] Greiner M Serodiagnostische Tests. Evaluierung und Interpretation in der Veterinärmedizin und anderen Fachgebieten 2003 Berlin, Heidelberg, New York, Hongkong, London, Mailand, Paris, Tokyo: Springer Verlag

[B46] Sackett DL Haynes RB Guyatt GH Tugwell P Clinical Epidemiology. A Basic Science for Clinical Medicine 1991 2 Boston, Toronto, London: Little, Brown and Company

[B47] Wirtz M Über das Problem fehlender Werte: Wie der Einfluss fehlender Informationen auf Analyseergebnisse entdeckt und reduziert werden kann. On the problem of Missing Data: How to Identify and Reduce the Impact of Missing Data on Findings of Data Analysis Rehabilitation 2004 43 109 115 10.1055/s-2003-814935 15100920

[B48] Church AH Waclawski J Designing and Using Organizational Surveys 1998 Hampshire, Vermont: Gower

[B49] Grosch JW Alterman T Petersen MR Murphy LR Worksite Health Promotion Programs in the U.S.: Factors Associated with Availability and Participation Am J Health Promot 1998 13 36 45 10186933 10.4278/0890-1171-13.1.36

[B50] Bandura A Self-efficacy: Toward a unifying theory of behavioral change Psychol Rev 1977 84 191 215 10.1037/0033-295X.84.2.191 847061

