# Supplementary material for: The Worksite Health Promotion Capacity Instrument (WHPCI): development, validation and approaches for determining companies' levels of health promotion capacity
Source: BMC Public Health. 2010 Sep 13;10:550. doi: 10.1186/1471-2458-10-550 (PMC2949769; doi:10.1186/1471-2458-10-550)
Supplement: Additional file 1 — Descriptive statistics for the two subscales of the Worksite Health Promotion Capacity Instrument and their items. The file contains a table in which the wording of the instrument's items is presented. Furthermore, the mean and standard deviation of each item and of the scales' sum scores are shown. [file 1471-2458-10-550-S1.DOC]

Table S1

Descriptive statistics for the two subscales of the Worksite Health Promotion Capacity Instrument and their items

| **Scale** | **Items** | **n** | **M** | **S.D.** |
| --- | --- | --- | --- | --- |
|  |  |  |  |  |
| Health Promotion Willingness | mean sum score | 517 | 5.66 | 2.24 |
|  | 1. The company’s management demonstrates a very strong willingness to actively promote employee health. | 519 | 5.75 | 2.88 |
|  | 2. There are influential people in our company who disregard the importance of health promotion entirely and who work against it.^1,2^ | 516 | 8.83 | 2.18 |
|  | 3. In our company, the prevailing opinion is that health is exclusively a personal matter.^1,3^ | 518 | 6.11 | 3.11 |
|  | 4. In our company, the subject of employee health promotion is often discussed. | 521 | 4.31 | 2.81 |
|  | 5. In our company, we firmly believe that we can carry out workplace health promotion efficiently. | 517 | 5.07 | 2.88 |
|  | 6. In our company, we are strongly convinced that, in general, it is possible to promote employee health. | 521 | 7.50 | 2.44 |
| Health Promotion Management | mean sum score | 517 | 2.33 | 2.68 |
|  | 7. Needs are systematically identified prior to the implementation of worksite health promotion measures. | 512 | 2.88 | 3.32 |
|  | 8. We define quantifiable goals for worksite health promotion. | 513 | 2.04 | 2.96 |
|  | 9. All worksite health promotion measures are constantly evaluated based on the defined goals. | 518 | 1.86 | 2.99 |
|  | 10. The worksite health promotion measures are modified based on evaluation results. | 506 | 2.90 | 3.44 |
|  | 11. Our company has a comprehensive health promotion program, in which individual measures are integrated. | 510 | 1.96 | 2.77 |

*Note*: M = mean sum score (range 0–10); S.D. = standard deviation

^1^ Re-coding was performed for the purpose of the analyses: do not agree at all (positive assessment) was coded as 10; agree completely (negative assessment) was coded as 0.

^2^ This item was excluded as a result of the exploratory factor analysis performed on the Health Promotion Willingness scale.

^3^ This item was excluded as a result of the reliability analysis performed on the Health Promotion Willingness scale.
