# Supplementary material for: The Worksite Health Promotion Capacity Instrument (WHPCI): development, validation and approaches for determining companies' levels of health promotion capacity
Source: BMC Public Health. 2010 Sep 13;10:550. doi: 10.1186/1471-2458-10-550 (PMC2949769; doi:10.1186/1471-2458-10-550)
Supplement: Additional file 2 — Factor loadings of the items of the Health Promotion Willingness and Health Promotion Management scales. The file contains a table in which the factor loadings of the items are presented as well as the eigenvalues and the results for the explained variance from three exploratory factor analyses. In addition, the corrected item-total correlation of the reliability analysis is given for each item. [file 1471-2458-10-550-S2.DOC]

Table S2

Factor loadings of the items of the Health Promotion Willingness and Health Promotion Management scales (n=517)

| **Scale** | **Item^1^** | **Factor loadings** | | | | | | | | **Corrected item-total correlation** | |
| --- | --- | --- | --- | --- | --- | --- | --- | --- | --- | --- | --- |
|  |  | **Model 1**^2^ | | **Model 2**^3^ | | **Model 3**^4^ | | | |  | |
|  |  | factor 1 | factor 2 | factor 1 | factor 2 | | factor 1 | | |  | |
| Health Promotion Willingness | 1 |  | 0.79 |  | 0.81 | | 0.85 | | 0.73 | | |
|  | 2 |  | 0.34 |  |  | |  | |  | | |
|  | 3^5^ |  | 0.64 |  | 0.64 | | 0.64 | | 0.48 | | |
|  | 4 |  | 0.61 |  | 0.68 | | 0.80 | | 0.65 | | |
|  | 5 |  | 0.63 |  | 0.69 | | 0.81 | | 0.67 | | |
|  | 6 |  | 0.77 |  | 0.79 | | 0.73 | | 0.58 | | |
| eigenvalue  explained variance |  |  | 2.64  24.0% |  | 2.90  29.0% | | 2.96 | | | |  |
|  |  |  |  |  |  |  | 59.1% | | | |  |
| Health Promotion Management |  |  |  |  |  | | | | | | |
|  | 7 | 0.83 |  | 0.82 |  | | | 0.86 | 0.78 | | |
|  | 8 | 0.90 |  | 0.89 |  | | | 0.90 | 0.83 | | |
|  | 9 | 0.91 |  | 0.91 |  | | | 0.85 | 0.85 | | |
|  | 10 | 0.82 |  | 0.80 |  | | | 0.80 | 0.77 | | |
|  | 11 | 0.77 |  | 0.75 |  | | | 0.91 | 0.71 | | |
| eigenvalue |  | 4.24 |  | 3.92 |  | | | 3.76 |  | | |
| explained variance |  | 38.5% |  | 39.2% |  | | | 75.1 |  | | |

^1^ For item wording, see Table 1.

^2^ All items of both scales in one exploratory factor analysis

^3^ Exploratory factor analysis following elimination of item 2

^4^ Separate exploratory factor analyses for each subscale

^5^ This item was removed from the scale as a result of the reliability analysis
